# Rehabilitation of Post-COVID-19 Musculoskeletal Sequelae in Geriatric Patients: A Case Series Study

**DOI:** 10.3390/ijerph192215350

**Published:** 2022-11-21

**Authors:** Mariana Cevei, Roxana Ramona Onofrei, Anamaria Gherle, Cristina Gug, Dorina Stoicanescu

**Affiliations:** 1 Psychoneuro Sciences and Rehabilitation Department, Faculty of Medicine & Pharmacy, University of Oradea, 410087 Oradea, Romania; 2 Department of Rehabilitation, Physical Medicine and Rheumatology, Research Center for Assessment of Human Motion, Functionality and Disability, “Victor Babes” University of Medicine and Pharmacy Timisoara, 300041 Timisoara, Romania; 3 Microscopic Morphology Department, “Victor Babes” University of Medicine and Pharmacy Timisoara, 300041 Timisoara, Romania

**Keywords:** medical rehabilitation, musculoskeletal sequelae, post-COVID-19

## Abstract

The musculoskeletal system is affected in over 40% of patients with Coronavirus disease 2019 (COVID-19). There is an increased need for post-acute rehabilitation after COVID-19, especially in elderly people with underlying health problems. The aim of this study was to evaluate the benefits of an early and goal-orientated rehabilitation program using combined approaches, robotic medical devices together with other rehabilitation techniques and therapies, in elderly people after acute COVID-19. Ninety-one patients (62.64 ± 14.21 years) previously diagnosed with severe SARS-CoV-2 infection were admitted to the Medical Rehabilitation Clinical Hospital Baile Felix, Romania, for medical rehabilitation, but only six patients (85.33 ± 3.07 years) met the inclusion criteria and participated in the study. The rehabilitation treatment was complex, performed over 4 weeks, and included combined approaches: exercise therapy, robotic gait training, occupational therapy, and massages. Activity and participation evaluation were performed using the Barthel Index and Functional Independence Measure for activities of daily living (ADLs). Assessments were performed at admission and discharge from the rehabilitation clinic. Lokomat patients’ reports revealed that the patients had improved motor control (with one exception). The measurement of functional ability revealed an improvement in most cases. This study presents some of the first data on outcomes of COVID-19 patients’ musculoskeletal rehabilitation in our country. Early complex medical rehabilitation improved functional independence and autonomy in ADLs in very old patients, post-COVID-19.

## 1. Introduction

Coronavirus disease 2019 (COVID-19) was declared a pandemic in March 2020 and has impacted the lives of many people. Severe acute respiratory syndrome coronavirus 2 (SARS-CoV-2) affects the human body on multiple levels, including the musculoskeletal system. Musculoskeletal symptoms such as myalgia, joint pain, and extreme fatigue are common [1,2,3,4]. Muscle weakness may be induced by immobility due to hospitalization for severe cases, iatrogenic use of steroids, or neuromuscular blocking agents. Other consequences of prolonged immobility include cardiorespiratory deconditioning, contractures, joint stiffness, postural instability, neuropathy, and pressure injuries. Acquired muscle weakness and muscle wasting resulting in musculoskeletal deconditioning contribute to a poor long-term functional outcome [5].

There is an increasing need for post-acute rehabilitation after COVID-19, especially in elderly people with underlying health problems. Medical rehabilitation is a key component in the continuous care of patients [6]. It helps to minimize or slow down the deconditioning consequences, the disabling effects of immobilization, joint stiffness, and pain, occurring frequently together especially in older patients, facilitating and improving long-term recovery and functional independence [7,8,9]. Rehabilitation programs need, therefore, to be tailored for each patient’s needs, aiming at the various deficits caused by COVID-19 and the pre-existing conditions and/or frailty. Each patient will need an individualized rehabilitation program, including breathlessness management, strength training, aerobic exercise, balance training, functional and vocational rehabilitation, and psychological support [10].

The application of robotic technologies in rehabilitation has progressed over the last few years as the number of people requiring medical rehabilitation increased rapidly [11]. Robotic medical devices are helpful for musculoskeletal therapy, restoring functionality and improving coordination, assisting disabled or older individuals. These rehabilitation robots provide opportunities to improve the quality of daily life of affected people [12].

A number of studies emphasize the need for early, intensive medical rehabilitation in patients after acute COVID-19, to reduce the functional disability duration. Even elderly patients, who usually have comorbidities/frailty, benefit from tailored geriatric rehabilitation treatment [13,14,15].

The aim of this study was to evaluate the benefits of an early and goal-orientated rehabilitation program, using combined approaches: robotic medical devices together with other rehabilitation techniques and therapies, in elderly people after acute COVID-19.

## 2. Materials and Methods

### 2.1. Participants

**Figure 1:**
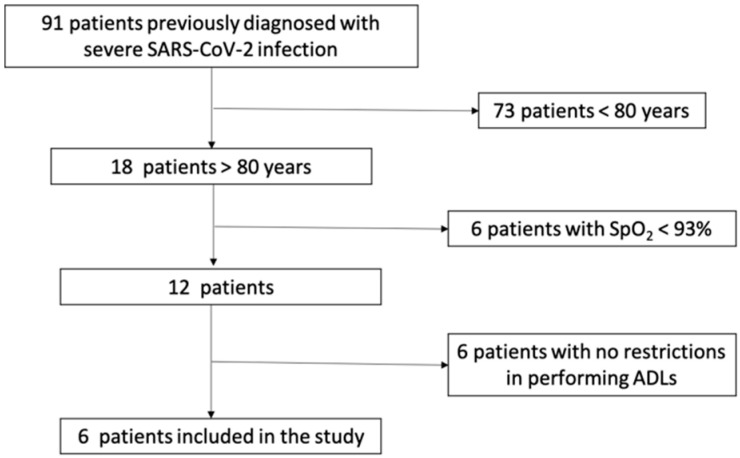
The flow chart of patients’ selection and inclusion criteria.

This case series study was conducted during the third wave of the COVID-19 pandemic, between February 2021 and May 2021. All patients previously diagnosed with severe SARS-CoV-2 infection admitted to the Medical Rehabilitation Clinical Hospital Băile Felix, Romania, for medical rehabilitation were invited to participate in the present study. Inclusion criteria were: (1) patients over 80 years old, previously diagnosed with severe SARS-CoV-2 infection, (2) with no clinical and biological signs of acute viral disease, (3) with loss of autonomy for activities of daily living 1 month after the diagnosis, (4) with musculoskeletal dysfunction, and (5) inability to walk. Patients with dyspnea at rest, O_2_ saturation under 93%, and cardiorespiratory instability were not included in the study (Figure 1). All patients signed the informed consent [16] and agreed to participate in the study. The study was approved by the Local Ethics Commission for Scientific Research of the Medical Rehabilitation Clinical Hospital Băile Felix, Romania (no 5001/14 May 2021).

### 2.2. Assessments

Demographic data and comorbidities were collected. COVID-19–specific data were collected retrospectively, from medical transfer documents—the number of hospitalization days in the COVID-19 hospital and several laboratory findings, including: ferritin, lactate dehydrogenase (LDH), C-reactive protein (CRP), fibrinogen in day 1 of admission in the COVID-19 hospital, day 7 of illness, and the day of admission to the rehabilitation clinic.

Vital signs of the patients were permanently monitored: body temperature, blood pressure, ventricular rate, respiration rate, blood oxygen level (SpO_2_), and 24 h urine volume.

A rating of the medical and psychiatric impairment was performed using the Cumulative Illness Rating Scale-Geriatric (CIRS-G) [17]. This validated scale evaluates 14 health domains, each rated from 0 (no problem) to 4 (an extremely severe problem). The scores for each domain are summed up, resulting in a total score range from 0–56, where 0 indicates no diseases and higher scores indicate higher severity. It allows identification of a few severe problems or multiple problems of mild to moderate severity, as it provides a rating for each organ system [17,18,19,20].

Musculoskeletal assessments—active and passive range of motion (ROM), handgrip strength test, muscle testing for girdles and limb muscles, stability, and balance assessments—were performed before starting the rehabilitation program and after the program was completed.

A universal goniometer was used to measure ROM. Patients were evaluated bilaterally for shoulder, hip, and knee joint ROM [21,22,23,24,25].

Grip strength was measured using a Jamar hand dynamometer, which is a validated test, considered to be the gold standard [26,27,28,29]. The maximum shown grip force can be 90 kg (200 lb). For the test, the patient had to be seated, with the shoulder adducted, the elbow flexed at 90°, the forearm in a neutral position and the wrist at 0 to 30° of extension and 0 to 15° of ulnar deviation. The values from three successive measurements for each hand tested were recorded. The average score was compared with the cut-off value of 27 kg in men and 16 kg in women [30,31].

Manual muscle testing using the Medical Research Council (MRC) scale [32,33] assesses muscle strength from grade 5 (normal power) to grade 0 (no contraction). The global muscle strength is quantified by the MRC-sum score, which represents the sum of all the obtained values [34].

Stability and balance assessments were measured using the evaluation tools for the trunk stability. The test was interpreted to be positive if the patients leaned to one or the other side.

Activity and participation evaluation were performed using the Barthel Index and Functional Independence Measure (FIM), for activities of daily living (ADL): basic activities of daily living (BADL) and instrumental activities of daily living (IADL). FIM is a validated assessment tool widely used in medical rehabilitation [35,36]. FIM high scores indicate patients with a higher level of independence, requiring a small amount of assistance. Two sub-scores, motor sub-score and cognitive sub-score, can be calculated. A maximum total FIM score of 126 points with a maximum motor sub-score of 91 points can be obtained [37,38]. The Barthel Index is a validated scale used to measure performance in ADL [39,40]. A higher Barthel score reflects a greater ability to function independently. Fully independent individuals score 100 points [41].

The contribution of rehabilitation therapy to regain functional autonomy (ADLs) was assessed using the FIM gain, Barthel Index Scores, and LOKOMAT parameters (speed; body weight support; distance change; duration change; L ROM; L FORCE) at hospital admission and discharge.

### 2.3. Rehabilitation Treatment

**Figure 2:**
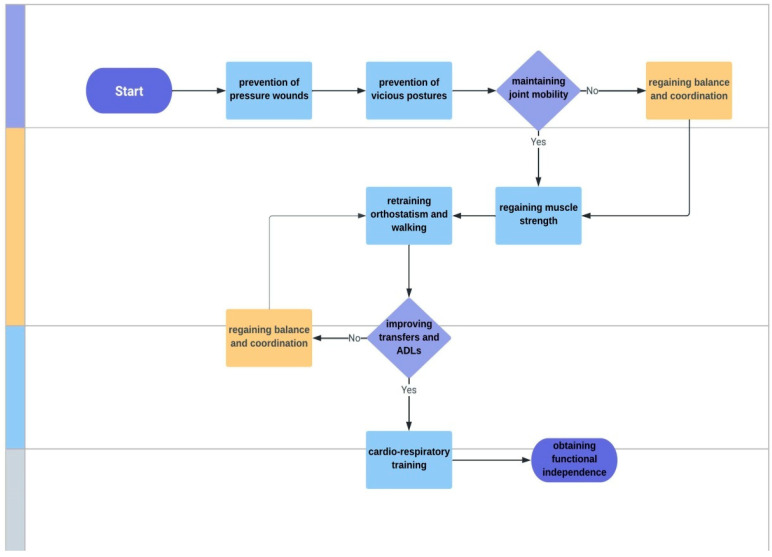
Main objectives of the rehabilitation program. ADLs—activities of daily living.

The main objectives of the rehabilitation treatment were: (1) prevention of pressure wounds; (2) prevention of vicious postures; (3) maintaining joint mobility; (4) regaining muscle strength, (5) retraining orthostatism and walking; (6) improving transfers and ADLs; (7) cardio-respiratory training; (8) obtaining functional independence; and (9) improving quality of life (Figure 2). The rehabilitation treatment was individualized based on joint mobility, muscle strength, balance, coordination, and gait.

The lifestyle recommendations were as follows: appropriate hydration (minimum 2 L of fluids/day); low-sodium diet (cases 2, 3, 4, 5); low glycemic index diet in patients with diabetes (cases 1, 2, 3); chronic liver disease diet (case 4); high calcium foods (cases 5, 6).

The rehabilitation treatment was complex and was performed over 4 weeks. It included combined approaches: exercise therapy, robotic gait training, occupational therapy, and massages.

#### 2.3.1. Exercise Therapy

Exercise therapy was performed twice a day for 30 min and started with muscle stretching and light muscle toning during the first week.

Passive mobilizations of the limbs were gently made, on the functional range of motion of the joint. The main aims of these mobilizations were to maintain a joint’s range of motion within functional limits, to soften the capsulo-ligament structures, to have a pumping effect on small muscle vessels and on the veno-lymphatic circulation, and to maintain the kinesthetic memory for the segments in question. Gradually, active-passive mobilization and then active mobilization were performed. Aerobic exercises with a gradual increase in intensity and duration of effort were introduced in the next stage of the physical therapy program.

Transfers reeducation was made using specific techniques. Patients had no neurological damage and most of the muscle strength was maintained. Three patients needed a wheelchair to reeducate transfers; the others used a rolling frame and crutches to start gait reeducation.

To increase muscle strength in the upper limbs, isotonic resistance active movements were performed. For the lower limbs, assisted active movements on the entire range of motion were performed followed by manual resistance movements and isometric exercises for toning the pelvic girdle. Strength training with progressive resistance was made from a sitting position and orthostatism, 8–12 repetitions for each muscle group, 1–3 muscle groups/unit of time, for 2 min.

Coordination and balance reeducation was performed with the use of assistive devices (crutch, walking stick, walking frame) and a Bobath ball; gait re-education used the treadmill in a straight and inclined plane.

#### 2.3.2. Occupational Therapy

Occupational therapy had the following objectives: to restore the active mobility, strength, and coordination in the upper and lower body; to acquire a maximum degree of functional independence in self-care; to establish a balance between rest, occupational, and recreational activities; and to improve ADLs and to increase the quality of life by optimizing the patient’s home environment to his/her individual abilities. The occupational therapy sessions were performed 30 min, 2 times/day. Passive pedaling exercises for upper and lower limbs were recommended in order to improve sitting stability. Verticalization (standing) was carried out gradually in order to avoid possible vegetative reactions. The patients’ attendants were instructed to correctly handle the patient both in transfers and in performing ADLs.

#### 2.3.3. Robotic Devices for Gait Reeducation

Robotic-assisted gait training was performed with the Lokomat^®^Pro (Hocoma, Switzerland). It provides what is known as automated locomotion therapy. Lokomat^®^Pro, a robotic medical device designed to be used in functional, intensive locomotor therapy, guides the patient based on a computer-controlled physiological gait pattern, providing precise, dynamic weight support. It has electrically operated walking braces, individually adjustable, which assist training on a treadmill and as a display for the visual feedback of performance and evaluation of gait activity. Training parameters (speed, weight support, range of motion, guiding force) can be adjusted to meet the patient’s requirements. The assessment tools used were L-FORCE (isometric force of flexion/extension muscle groups in hip and knee joints) and L-ROM (range of motion of flexion/extension in hip and knee joints). The system stimulates the muscle fibers by intensely repetitive movements associated with visual, auditory, and tactile feedback; it trains the lower body, initially providing body weight support for the patient, which eventually decreases gradually allowing the patient to perform a physiological walking pattern. The first session’s duration was 15 min, progressively increasing the time, reaching 30 min daily, and progressively increasing the speed of movement.

#### 2.3.4. Massage

All patients included in the study received massage therapy daily, for 20 min. Effleurage technique for the cervical, dorsal, and lumbar spine was recommended, aiming to improve muscle relaxation, reduce the severity of muscle soreness, soften tender and trigger points, and to have a general sedative effect. A lower limbs massage was performed in case 2 and case 6, using two basic strokes of massage, effleurage, and petrissage, targeting circulation improvement, facilitating an increase of mobility of the joints and soft tissues, and reducing edema [42,43,44,45].

### 2.4. Statistical Analysis

Data were tested for normality with a Kolmogorov–Smirnov test and presented as mean ± SD. Paired t-tests were performed to identify the differences between results at admission and discharge. A repeated measures ANOVA test was used to identify the differences in ferritin, LDH, fibrinogen, and CRP levels. A *p*-value < 0.05 was considered significant.

## 3. Results

Ninety-one patients (62.64 ± 14.21 years) were admitted to the Medical Rehabilitation Clinical Hospital Baile Felix, Romania for medical rehabilitation after acute COVID-19, during the third wave. Of these, six patients aged over 80 years (mean age 85.33 ± 3.07 years; four males) met the eligibility criteria. They were all previously diagnosed with severe SARS-CoV-2 infection. Patients’ characteristics, according to data from their medical records, are presented in Table 1.

The assessment findings during hospitalization in a COVID-19 hospital (assessment 1: day 1 admission in a COVID-19 hospital, assessment 2: day 7 in a COVID-19 hospital, and assessment 3: the lab values from day 1 admission in the rehabilitation hospital) for ferritin, LDH, fibrinogen, CRP are shown in Table 2.

Serum ferritin levels remained elevated even during hospitalization in the medical rehabilitation hospital (assessment 3) in all six patients. Levels above the upper limit of normal for serum LDH, considered predictive for the severity of COVID-19 [46], were noted in all the analyzed cases during hospitalization in the COVID-19 hospital. Case 1 had progressively increased levels, with the highest value at admission in our hospital (725 U/L). A slight increase was also noted in case 6. The inflammatory syndrome remitted almost completely, the fibrinogen values being within normal ranges at admission in the rehabilitation hospital, and the CRP decreased significantly, the highest value being recorded in case 4 (14.5 mg).

The mean handgrip strength was 12.72 ± 3.81 kg for the right hand and 13.6 ± 5.93 kg for the left hand (Table 3). In men, hand grip strength was below the cut-off values at both baseline and final assessments, bilaterally. However, a slight improvement was noted. Mean values of right-hand grip strength at admission and discharge were 12.24 ± 4.70 and 18.08 ± 4.04, respectively (*p* = 0.05). Mean values of left-hand grip strength at admission and discharge were 10.99 ± 2.95 and 11.49 ± 3.9393, respectively (*p* = 0.39). In case 5, the baseline assessment revealed that hand grip strength was below the cut-off value, bilaterally, and above it, bilaterally, after the rehabilitation program was completed. Right-hand grip strength was below the cut-off threshold and returned to normal values after medical rehabilitation in case 6.

Cases 1 and 6 had MRC grade 5 and the other cases had grade 4.

Descriptions of Lokomat patients’ reports regarding changes in the first/last sessions are presented in Table 3 and Table 4.

With the exception of case 3, there was an improvement in speed, body weight support, and the covered distance and duration, all assessed at each session. The device settings show the average percent change (increase/decrease) of these variables, assessed daily.

There was an improvement in hip and knee mobility as a result of robotic gait reeducation (Table 3). The data provided by Lokomat for L-ROM showed that the range of motion increased in the hip and knee joints, and there was a remarkable improvement of the right hip extension. The most spectacular increase of hip and knee ROM was noticed in case 1; at the opposite pole, in case 3, the very low ROM baseline values were maintained throughout the rehabilitation program. In case 4, even if ROM, which was slightly decreased compared to age ranges, changed only by a few degrees during hospitalization, the benefit for ambulation and other activities is undeniable.

Hip and knee muscles strengthening, for both flexors and extensors, led to an increase of the patient’s contribution to the mobilization of the lower limbs in all cases. The most important gain was noticed for the right hip flexor muscles.

Measurement of functional ability using Barthel Index and FIM scores assessed at admission and discharge from the rehabilitation clinic revealed an improvement in five cases (Table 3). Functional assessment with FIM revealed the reduction of functional dependence in five cases (case 3 was stationary); the most notable functional gain was seen in case 6, which showed minimal dependence after 30 days of rehabilitation. The final assessment revealed that cases 1 and 4 were moderately dependent after initially being maximally dependent. Case 2 evolved from moderate dependence to minimal dependence. Unlike the motor subtotal score, which had an upward trend of improving daily activities consequently reducing functional dependence, the cognitive subtotal score remained unchanged.

## 4. Discussion

This study assessed the benefits of an early and goal-orientated rehabilitation program, using combined approaches: robotic medical devices together with other rehabilitation techniques and therapies, in several post-COVID-19 cases that met the inclusion criteria. The obtained results revealed that functional independence has been improved, with increased mobility, ability to carry out transfers, autonomous walking, as well as patients’ self-care capacity.

Our study included only six elderly people (over 80 years old, four men), mainly because there were several limiting factors regarding the eligibility of the patients included in the study, such as: (1) age of the patients at the moment of the study, (2) multiple comorbidities, (3) and cardiopulmonary decompensation present in some patients. By the end of the fourth week of complex rehabilitation, five out of the six patients showed improvements in muscle strength, coordination and balance, and reeducation of transfers.

COVID-19 disproportionately endangers older individuals, especially those with pre-existing comorbidities [47]. The minimum CIRS-G score was 10 in our cases, with a mean of 16.33 ± 8.68 and a maximum of 32. As seen in our patients, Steinmeyer et al. reported that old patients represent a very heterogeneous group. They measured patient comorbidities using the CIRS-G scale, obtaining a total score of 12.3 ± 5.1, and concluded that assessing an older patient’s health status based on multidisciplinary geriatric assessments is important in identifying those most at risk of negative health outcomes [48]. Moloney et al. also rated total comorbidity disease burden in older persons using the CIRS-G scale and over one third of patients had a CIRS-G score greater than 10 [49]. It is worth mentioning that chronological age does not always reflect the physiological reserve to overcome acute events [50].

Serum ferritin levels were elevated in all six patients admitted to medical rehabilitation. Many COVID-19 patients have hypoxia that may be due to various affected processes and disturbed iron metabolism could be one of them. Data show that these patients tend to present pathologically increased levels of ferritin. Mean pooled ferritin levels were found to be higher in older individuals, males, people with severe forms of the disease, and hypertensive patients [51,52]. Three patients had higher levels of LDH when admitted to our hospital. Elevated LDH levels were associated with a severe form of the disease [46].

Prolonged immobilization has various consequences on the neuro-muscular system. Muscle mass and function are impaired during long-term hospitalization. COVID-19 patients, especially those with severe forms, require many days of hospital care. The hospital length of stay of our patients ranged between 12–22 days, with a mean of 17 days. Earlier reports have shown that the peripheral muscle strength decreases by approximately 20% every week of bed rest in the hospital [53,54]. Gruther et al. [55] found a negative correlation between loss of muscle mass and length of hospital stay. Loss of muscle mass seemed to be particularly marked during the first 2–3 weeks of immobilization. Muscular atrophy induced during hospitalization for severe forms of COVID-19 can lead to muscular dysfunction that persists for several months [56]. Severe muscle weakness, extreme fatigue, impaired functioning with reduced mobility and declined ADLs, and neuro-psychological problems were long-term consequences observed in COVID-19 patients hospitalized in intensive care units [57]. Older patients will not only have the consequences of prolonged immobilization in severe forms of COVID-19, but also deficits due to the comorbidities that each one has. As a result, a multidisciplinary team approach is recommended in the post-acute rehabilitation of COVID-19 patients [10].

Impairments occurring post-COVID-19 must be the targets of medical rehabilitation in medium-and long-term monitoring. Early rehabilitation can positively affect functional status. When developing rehabilitation strategies, it must be taken into account that many patients, especially the older ones, do not tolerate early intense physical activities, showing rapid oxygen desaturation [53,58]. Therefore, in these cases, physical activities should slowly and progressively increase in intensity. A 6-week rehabilitation program aiming to improve physical capacity proved to be effective [53].

After 4 weeks of medical rehabilitation in our hospital, which included various approaches (exercise therapy, robotic gait training, occupational therapy, massages), five patients improved their muscle strength, coordination, balance, and transfers. Handgrip strength in the dominant (right) limb improved statistically significantly, which contributes to the improvement of distal motor control and the performance of ADL that involve the hand. Low grip strength was noticed to be a predictor for severe COVID-19 disease [59]. A systematic review showed that exercise training increased grip strength and improved the functional capacity and quality of life [60].

Another systematic review performed by Goodwin and collaborators reported that even the existing evidence for rehabilitation after being discharged from the hospital following an ICU admission for COVID-19 is inconclusive, as patients receiving rehabilitation valued it [61]. Another previous study has suggested that an intensive exercise program of 90 min, five days/week, did not appear to improve physical outcomes at 6 months compared with the standard exercise program of 30 min [62].

The objective of gait reeducation with the aid of the robotic devices is to improve muscle strength, which is why we have not seen major changes when evaluating mobility, except for hip extension. Muscle strength assessment showed that almost all the studied parameters (except the left knee extension) improved. An improvement was noticed for the right hip flexion. We performed a PubMed search using the following keywords— Lokomat, medical rehabilitation, and post-COVID-19—to compare our data with those from the previous research reported in the literature regarding the use of Lokomat for medical rehabilitation of people after acute COVID-19. We did not find any relevant results.

With the exception of case 3, different degrees of functional autonomy gain were noticed in our patients: minimal assistance (cases 2, 5, 6) and moderate assistance (cases 1, 4) at discharge. The level of functional independence measured with the Barthel Index at the time of discharge revealed severe dependence (cases 1, 2, 4) and minimal dependence (cases 5, 6). There was a considerable improvement of functional outcome after rehabilitation. Motor FIM gain and Barthel score improvement were remarkable in case 6, a female patient aged 88. The FIM and Barthel scores at discharge showed an increase in the ability to perform ADL, with the consequent improvement of functional independence, but patients were not fully independent. This might be due to their age as well as due to the comorbidities they have. Studies aiming to examine associations between body mass index, age, gender, and functional limitations revealed that men reported fewer limitations than women, and women aged over 75 years and men over 90 years showed high difficulty walking, climbing stairs, and bending and carrying loads, with gender being an important predictor of functional limitations [63,64].

Patient-tailored rehabilitation was also endorsed by Ferraro et al., concluding that this is mandatory for reducing fatigue and improving functional outcome [65]. Motor-based FIM score improvements after COVID-19–positive individual rehabilitation were also reported by Journeay et al. They noticed a relatively rapid improvement in mobility and motor function in many cases [66]. A study performed by Fundarò et al. indicated that Lokomat training significantly improved the parameters provided by the Lokomat itself, as well as global and motor FIM scores [67].

Our study has some limitations. The small number of patients included in the study and the absence of a control group are some of these limitations. The case series design of this study is not the strongest source of evidence. Changes observed might be casual or due to the natural history of the disease. Karasu et al. [68] observed significant improvement in the muscle strength, physical performance, and musculoskeletal symptoms of patients with COVID-19 over time, but the physical performance did not reach normal values, concluding that post-COVID-19 rehabilitation programs are needed. Further studies including a larger number of patients with a longer follow-up period are needed in order to evaluate the rehabilitation interventions needed to reduce COVID-19–related disability, especially in very old adults. COVID-19 is a new disease that has affected a huge number of individuals; therefore, we consider that the strength of this study consists of providing descriptive information and building knowledge in this field.

## 5. Conclusions

This study presents some of the first data on outcomes of COVID-19 patients’ musculoskeletal rehabilitation in Romania. Handgrip strength improved bilaterally, especially in the dominant (right) limb, contributing to the performance of ADLs that involve the hand. The overall force of the lower part of the body was significantly increased after using the robotic rehabilitation devices, helping the patients to maximize the function, autonomy, and quality of life. Early complex medical rehabilitation using a progressive treatment plan, which focused on function, disability, and return to participation in society, improved functional independence and autonomy in ADLs in very old patients, post-COVID-19.

## Figures and Tables

**Table 1:** Patients’ characteristics.

Case	Gender	Age (Years)	Hospitalization Days for COVID-19	Comorbidities	CIRS-G Score	MRC Grade
1	M	88	22	Type 2 diabetes requiring insulin Mild dementia	10	5
2	M	81	12	Type 2 diabetes not requiring insulin Hypertension stage II with high cardiovascular risk History of myocardial infarction Mild dementia	13	4
3	M	82	15	Type 2 diabetes requiring insulin COVID-19–related myocarditis Hypertension stage II with high cardio-vascular risk Class 2 obesity Alzheimer disease Heart failure NYHA class II	21	4
4	M	86	16	Hypertension stage II with high cardio-vascular risk Chronic hepatitis Post-stroke sequelae Chronic kidney disease Ileostomy Moderate dementia Moderate anemia, secondary to COVID-19 infection Prostate adenoma	32	4
5	F	87	16	Hypertension stage II with high cardio-vascular risk Asthma Status post left total hip arthroplasty Stage 1 hip osteoarthritis Postmenopausal osteoporosis Moderate anemia, secondary to COVID-19 infection Mild dementia	10	4
6	F	88	21	Post-COVID-19 deconditioning syndrome. Moderate anemia, secondary to COVID-19 infection Type II osteoporosis Severe sarcopenia Right ovarian cyst Umbilical hernia	12	5
		85.33 ± 3.07	17 ± 3.79		16.33 ± 8.68	4 [4.5]

CIRS-G: Cumulative Illness Rating Scale-Geriatric. MRC: Medical Research Council.

**Table 2:** Patients’ characteristics according to the serum levels of ferritin, LDH, fibrinogen, and CRP.

Case	Ferritin (NV: M:15–200 ng/mL; F: 12–150 ng/mL)			LDH (NV: 135–225 U/L)			Fibrinogen (NV: 200–400 mg/dL)			CRP Quantitative (NV: <10 mg)		
	Assessment			Assessment			Assessment			Assessment		
	1	2	3	1	2	3	1	2	3	1	2	3
1	1042.3	876	2391	233	468	725	372	468	353	21.59	17.35	13.4
2	537	570	656.5	256	196	178	627	392	261	52.43	10.87	0.63
3	1388.5	943	930.7	543	498	255	488	339	255	92.04	34.55	5.23
4	945	678	530	423	297	130	622	419	213.2	142.43	39.88	14.5
5	326	333.4	406.4	455	262	199	540	372	260	480	110	1.2
6	406.6	932	1498.8	230	285	297	574	477.2	376	46.89	20.65	2.44
Mean ± SD	774.23 ± 417.29	722.06 ± 241.95	1068.9 ± 755.14	356.67 ± 134.35	334.33 ± 120.7	297.33 ± 217.53	537.16 ± 96.26	411.2 ± 54.33	286.36 ± 63.48	139.23 ± 172.19	38.88 ± 36.49	6.23 ± 6.19
*p*-Value	0.35			0.8			0.001			0.07		

NV—normal values; assessment 1: day 1—admission in a COVID-19 hospital; assessment 2: day 7—in a COVID-19 hospital; assessment 3: day 1—admission in the rehabilitation hospital; LDH—lactate dehydrogenase; CRP—C reactive protein.

**Table 3:** Patients’ assessment results.

Assessments	Case 1		Case 2		Case 3		Case 4		Case 5		Case 6		Mean ± SD		*p*-Value
	Ad	Di	Ad	Di	Ad	Di	Ad	Di	Ad	Di	Ad	Di	Ad	Di	
Grip strength (kg)															
Grip strength right hand	17.33	21.00	14.66	21.33	10.33	12.66	6.66	17.33	15.00	21.00	12.33	17.33	12.72 ± 3.81	18.44 ± 3.38	0.004
Grip strength left hand	15.33	17.33	10.00	14.66	8.66	10.00	10.00	19.40	13.00	17.00	24.66	27.00	13.61 ± 5.93	17.56 ± 5.62	0.021
L ROM (degrees)															
Hip flexion right	27	51	17	59	17	18	45	50	30	46	24	31	26.67 ± 10.41	42.5 ± 15.13	0.052
Hip flexion left	24	46	24	64	19	19	45	52	40	47	9	16	26.83 ± 13.41	40.67 ± 19.08	0.06
Hip extension right	10	14	5	10	0	0	10	10	5	14	14	17	7.33 ± 4.97	10.83 ± 5.95	0.052
Hip extension left	10	13	6	12	0	0	11	11	0	7	0	5	4.50 ± 5.21	8 ± 4.98	0.03
Knee flexion right	55	60	75	78	20	20	62	60	27	52	52	53	48.50 ± 21.04	53.83 ± 19.02	0.24
Knee flexion left	65	75	79	80	19	19	63	60	25	50	30	36	46.83 ± 25.14	53.33 ± 23.30	0.17
Knee extension right	5	5	10	10	0	0	12	12	6	12	5	5	6.33 ± 4.23	7.33 ± 4.80	0.36
Knee extension left	5	5	9	10	0	0	13	10	7	9	5	5	6.50 ± 4.37	6.50 ± 3.94	1
L FORCE (Nm/degree)															
Hip flexion right	29	40	46	65	0.7	10	46	68	48	61	27	46	32.78 ± 18.21	48.33 ± 21.76	0.0007
Hip flexion left	29	40	46	65	0.4	12	46	68	18	59	16	47	25.9 ± 18.04	48.50 ± 20.83	0.005
Hip extension right	28	39	28	44	0.1	10	38	52	28	49	14	48	22.68 ± 13.45	40.33 ± 15.52	0.004
Hip extension left	28	39	28	44	0.1	14	38	52	32	58	22	54	24.68 ± 13.14	43.50 ± 16.02	0.002
FIM Score															
MSS	16	45	37	62	15	17	18	42	76	83	16	64	29.67 ± 24.18	57.17 ± 22.71	0.02
CSS	24	24	26	27	7	7	16	17	33	33	18	25	20.67 ± 9.03	22.17 ± 9.04	0.23
Total	40	69	63	89	22	24	36	63	109	116	34	89	50.67 ± 31.57	75.00 ± 31.16	0.02
Barthel Score	15	40	15	50	0	0	10	30	65	95	5	90	18.33 ± 23.59	50.83 ± 36.39	0.09

Ad—admission; Di—discharge; SD—standard deviation. L—ROM (range of motion of flexion/extension in hip and knee joints). L-FORCE (isometric force of flexion/extension muscle groups in hip and knee joints).

**Table 4:** Lokomat assessment: changes in the first/last sessions.

Case	1	2	3	4	5	6
Speed change (km/h)	+158%	+49%	+5%	+35%	+52%	+30%
Body weight support (kg)	+47%	+48%	+5%	+35%	+17%	+33%
Distance change (meters)	+381%	+344%	0%	+221%	+195%	+76%
Duration change (minutes)	+99%	+246%	0%	+140%	+99%	+39%
